# Video-tactile pneumatic sensor for soft tissue elastic modulus estimation

**DOI:** 10.1186/s12938-017-0390-3

**Published:** 2017-08-01

**Authors:** M. M. Gubenko, A. V. Morozov, A. N. Lyubicheva, I. G. Goryacheva, M. Z. Dosaev, M.-Sh. Ju, Ch.‐H. Yeh, F.-Ch. Su

**Affiliations:** 1 A. Ishlinsky Institute for Problems in Mechanics RAS, Moscow, Russia; 2 Institute of Mechanics, Lomonosov Moscow State University, Moscow, Russia; 3 Department of Mechanical Engineering, National Cheng Kung University, Tainan City, Taiwan; 4 Medical Device Innovation Center, National Cheng Kung University, Tainan, Taiwan; 5 Department of Biomedical Engineering, National Cheng Kung University, Tainan City, Taiwan

**Keywords:** Indentation, Laparoscopy, Minimally invasive surgery, Tactile sensor, Young’s modulus

## Abstract

**Background:**

A new sensor for estimating elasticity of soft tissues such as a liver was developed for minimally invasive surgery application.

**Methods:**

By measuring deformation and adjusting internal pressure of the pneumatic sensor head, the sensor can be used to do palpation (indentation) of tissues with wide range of stiffness. A video camera installed within the sensor shell is used to register the radius of the contact area. Based on finite element model simulations and the measured data, elastic modulus of the indented soft tissue can be calculated.

**Results and conclusions:**

Three phantom materials, namely plastic, silicone and gelatin, with varied stiffness were tested. The experimental results demonstrated that the new sensor can obtain highly reliable data with error less than 5%. The new sensor might be served as an instrument in laparoscopic surgery for diagnosis of pathological tissues or internal organs.

## Background

Ongoing improvement of medical equipment enables an invention of new less traumatic surgical instruments called minimally invasive surgery tools. One of the first operations using minimally invasive surgery tools was performed in 1901 by Ott, who is known as one of the pioneers of laparoscopy [1]. Nowadays, laparoscopic surgery in which manipulation of internal organs of abdominal or pelvic cavity through small incisions not greater than 1.5 cm in diameter is highly popular according to overall surgery statistics. The main tools used to perform this kind of operation are non-automated laparoscopic tools and robotic surgical system such as the Da Vinci system. Sometimes, during an operation, it is necessary to determine elastic properties of tissues and tissue pathologies, for instance, tumors. During open surgery the surgeons determine the hardness of tissue using a simple and informative method—palpation. However, during a laparoscopic operation it is impossible to perform manual palpation. In the past a number of devices to determine stiffness of soft tissue have been developed. Many approaches such as physical wave methods—magnetic resonance elastography [2, 3] and ultrasound scans [4, 5]—as well as sensor [6] for relative soft tissue compliance have been suggested. The main function of these devices is to find malignant inclusions within the soft tissues and define their size.

Another method [7], based on measuring the contact pressure distribution in the area of interaction between cylindrical sensor and tissue surface, allows a map of stiffness to be built after performing series of tests on the target area. If a tumor appears to be in the examined organ, its location and size can be seen on the map.

A device can be used to estimate a density of tissues and the data can be transmitted to a tactile display. Palpating the display, a surgeon can feel a density of examined tissue [8]. Another sensor system is proposed to measure pressure and deformation of a tissue surface [9]. A reacting force of the tissue exerted to the spherical tip of the sensor is counterbalanced by an air pressure inside the sensor and it can be operated during manipulations.

However, the above mentioned methods do not quantitatively determine mechanical properties of the internal organs of a patient during a laparoscopic operation. Information about local mechanical properties of soft tissues is necessary for assessing its deviation from that of healthy tissue and for an instant detection of tumors inside an examined organ. New methods have been developed to estimate the mechanical properties of human internal organs. A device proposed for a minimally invasive surgery operation allows determination of the stiffness of tissue [10]. It contains a miniature sensor having a cavity with adjustable internal pressure. When the sensor comes in contact with an examined organ, the surface of the tissue is aspirated into the cavity through an aperture at the end of a cylindrical device. Both tissue deformation and applied air pressure inside the cavity are measured. A mathematical model of tissue response is employed to estimate the viscoelastic properties of the tissue and to discern between mechanical behavior of healthy and diseased organs.

The goal of research [11, 12] was to develop a new sensor that performs not only determining Young’s modulus of soft tissues, but also examination of the presence and location of possible tumors. The device described in [12] has a soft silicone tip for safe indentation of biological tissues. However, the proposed sensor is too large for using during laparoscopic operation. Reduction in size of such sensor faces many different problems, such as accuracy of image processing, unpredictable behavior of elastic sensor head and so on.

In this paper we present a miniaturized tactile video sensor, equipped with an optical sensor that allowed measuring displacement of central point of the sensor head, and an air pump that can adjust pneumatic pressure inside the sensor head. Varying an additional air pressure allows us to change sensitivity of the system. Deformation of the tip, contact area and internal pneumatic pressure of the sensor head are measured during the indentation. An Young’s modulus of tissue in consideration is obtained by fitting the experimental data to results of calculation of a finite element model that simulates the indentation process. Tumor detection technique is a subject of our further studies.

## Methods

### Sensor design and measurement technique

**Fig. 1:**
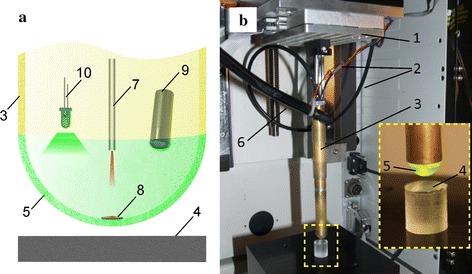
**a** Schematic and **b** photograph of the sensor installed in a UMT, where *1* is the load cell, *2* commutation cords of the video camera and backlight, *3* cylindrical tube, *4* sample, *5* silicone shell of the sensor head, *6* compressed air supply, *7* optical proximity sensor, *8* mirror, *9* video camera, *10* LED

Three phantom materials with varied stiffness, namely rigid plastic, silicone and gelatin were tested. Gelatin phantom sample was used for imitation of soft biological tissues. The miniature video-tactile pneumatic sensor was developed to measure the contact mechanical characteristics of soft biological tissues. The schematic diagram of the sensor is shown in Fig. 1a. Figure 1b shows the device mounted on a universal micro-mechanical tester (UMT) for calibration of the sensor system.

The video-tactile pneumatic sensor is based on an airtight cylinder attached at one end to a load cell. A soft silicone shell is fitted at the other end of the cylinder. The cylinder contains a video camera, a non-contact optical proximity sensor, and a light-emitting diode (LED). The LED serves as a light source that adjusts the amount of light inside the silicone shell to enable visualization of the optical image obtained by the video camera. The optical proximity sensor can measure a displacement of the shell tip. For this purpose a miniature mirror (diameter 1.5 mm) is glued centrally on inner surface of the shell.

During the experiments, the central part of the shell may be bended inwards when indenting a soft tissue and resulting in a ring shaped instead of circular contact area. To avoid this effect and to ensure a circular contact area, a compressed air was supplied to maintain a constant additional internal pressure measured with a manometer. This adjustable additional pressure inside the silicone head can tune the stiffness of the video tactile sensor and the sensitivity of sensor system. This approach enables measuring an Young’s modulus of soft tissues over a wide range.

**Fig. 2:**
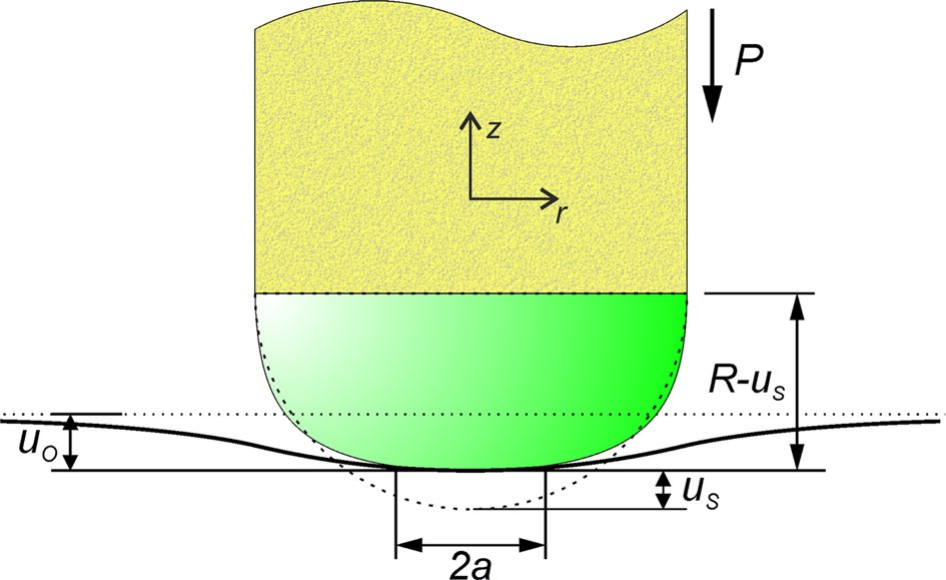
Scheme of the sensor shell (of radius *R*, mm) and the sample surface contact, where the *dotted lines* indicate un-deformed state at initial contact

During the indentation, two contact parameters were measured: the contact area radius *a* (mm) and the displacement of the central point of the shell *u*_*s*_ (mm) relative to the cylindrical tube of the sensor, which is obtained by the optical proximity sensor (Fig. 2).

**Fig. 3:**
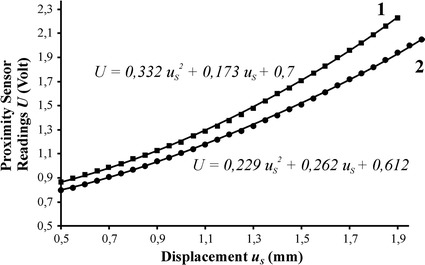
Dependence of optical sensor readings *U* (volt) on displacement of shell central point under various air pressure conditions: *1* 6 kPa, *2* 15 kPa

To calibrate the proximity sensor, indentation tests were first performed on the plastic sample, which is a rigid body. The origin *O* of the cylindrical coordinate system *Orφz* coincides with the shell center for the non-deformed condition, and the *Oz* axis goes up along the symmetry axis. The displacement *u*_*s*_ measured during tests relates to the displacement *h* of the cylindrical tube in the absolute coordinates, according to the following equation: $h = u_{S} + u_{0}$, where $u_{0}$ is the displacement of the central contact point of examined sample surface ($u_{0} = 0$ in case of a rigid sample). The experimental data was approximated by a parabolic function $U = Au_{S}^{2} + Bu_{S} + C$, where *U* represents the output voltage of the proximity sensor, *u*_*s*_ is measured in millimeters, and *A, B, C* are coefficients to be determined. The obtained functions were used for calibrating the optical proximity sensor under various pressure conditions inside the video-tactile sensor head. The calibration results for two internal pneumatic pressures are shown in Fig. 3.

**Fig. 4:**
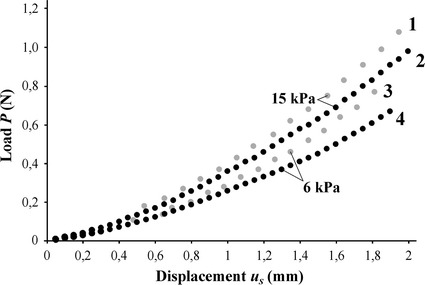
Dependence of normal load *P* applied to the sensor on the shell displacement *u*_*s*_ for materials with varying stiffness (*gray markers 1*, *3*—a soft silicone sample, *black markers 2*, *4—*a rigid sample) and varying air pressure: *1*, *2*—15 kPa; *3*, *4*—6 kPa

The normal load *P* applied to the video-tactile pneumatic sensor was plotted against the displacement *u*_*s*_ of the central shell point (Fig. 4) to illustrate sensitivity of the device. It is shown that varying the pressure inside the cylindrical tube affects the dependence of the load on the central point displacement.

**Fig. 5:**
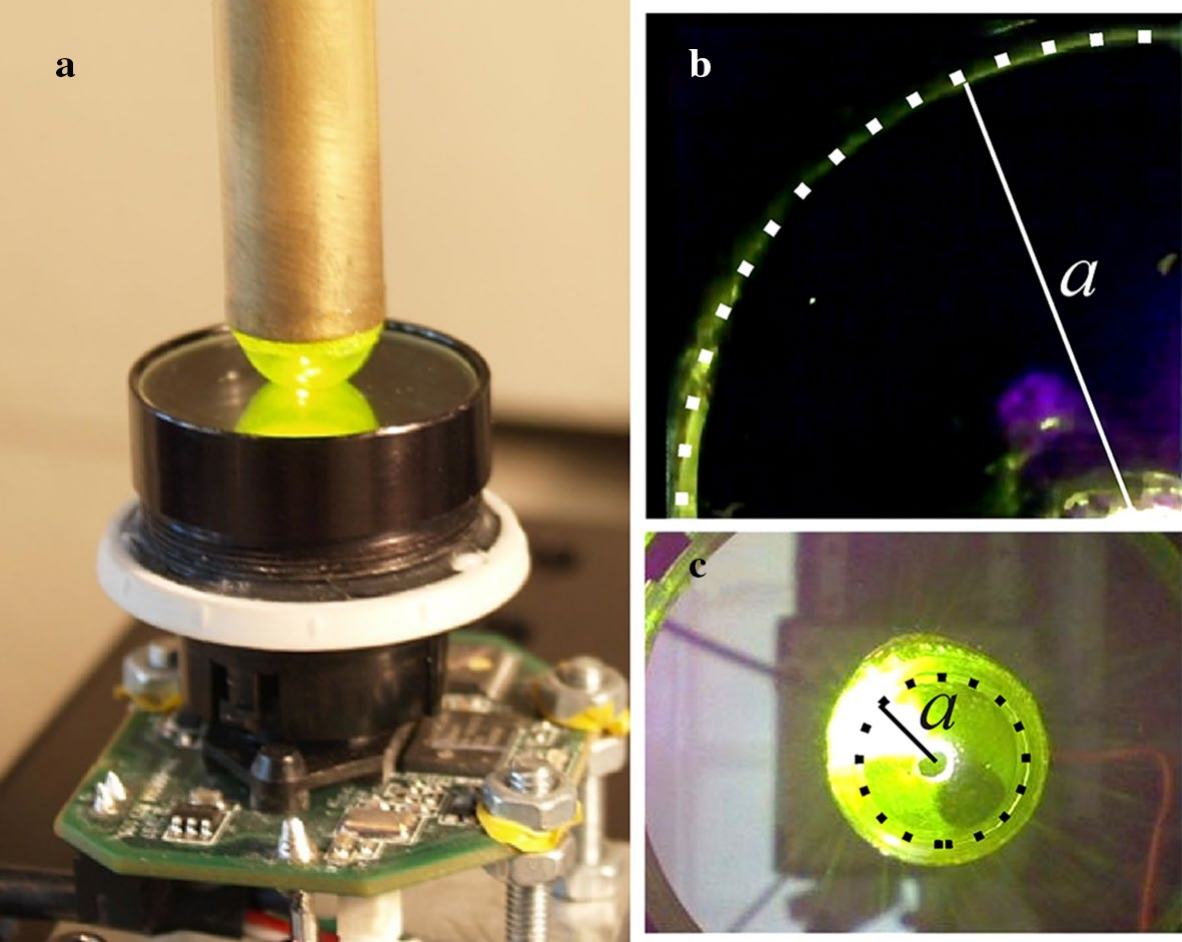
**a** The sensor indenting the glass installed on the reference camcorder. Images of the contact area from the built-in video camera (**b**) and from the reference camcorder (**c**). *Black* and *white markers* point the boundary of the contact area

The built-in video camera is used for registration of the contact area between the sensor head and tissue in study. To ensure accuracy of measurements of the contact area this camera was calibrated using a reference external camcorder (Fig. 5a). During indentation of glass cover of the external camera, the contact area can be observed from both camcorders simultaneously: from the internal camera of the device and from the reference pre-calibrated camera. To determine the contact area on the image from the reference camcorder its shape was approximated by circles as it can be seen on Fig. 5c (the contact area is assumed to be circular). After that, the internal camera was calibrated by doing the same procedure (Fig. 5b) for area with a known radius. In future, the whole procedures of calibrating the camera and measuring the contact radius can be done automatically using digital image processing algorithms.

### Finite element model for elastic modulus estimation

A finite element model for estimating an elastic modulus of the examined tissue was constructed. Due to the large deformations, the contact between the sensor and soft tissues is a geometrically non-linear contact problem that was solved numerically.

The transparent shell of the sensor tip is made of polydimethylsiloxane (PDMS). This material demonstrates elastic behavior for the selected load levels. Young’s modulus of PDMS is also measured by the spherical indentation test in previous study [12] and appears to be $E_{1} = 2300{\text{ kPa}}$, while Poisson’s ratio is assumed to be $\nu_{1} = 0.49$ since PDMS is close to the incompressible material [13] (hereinafter subscript 1 is used for indenter and subscript 2 is used for sample material).

Three samples, namely plastic cylinder, silicone cylinder and gelatin phantom (20 per cents gelatin in water) were tested. All three materials were considered as homogeneous and isotropic. The plastic was used as a standard sample for calibrating the sensor. Silicone sample was made from the same material as the sensor head. We assumed this material as linear elastic, with Young’s modulus *E*_2_ = *E*_1_. Calculations are also made under assumption that gelatin phantom is an incompressible material, and its elastic modulus should be determined by fitting the experimental data to model simulation data. As indicated in [14], modeling of soft biological tissue using linear elastic incompressible material ν_2_ = 0.499 is allowed when the indenting speed is small. Another important issue for each contact pair is the friction coefficient, the value of which will be discussed when formulating the boundary conditions.

To ensure reliable modeling of a contact interaction between the sample and the shell with pneumatic pressure inside, the problem was divided into two stages. The objective of stage 1 is to determine the shape and stress–strain curve of the shell under internal air pressure before contact interaction. The objective of stage 2 is to determine the contact mechanics.

**Fig. 6:**
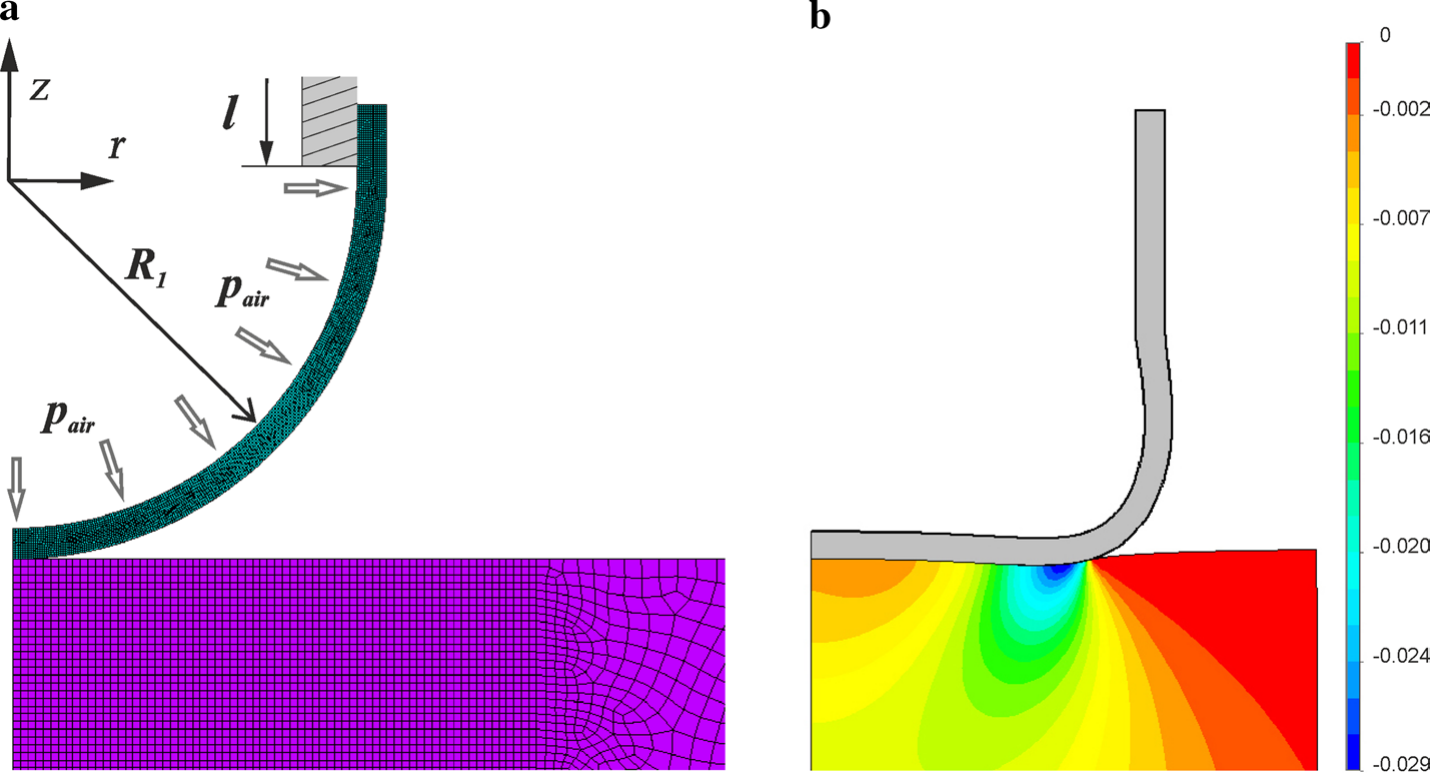
**a** Contact scheme and a fragment of the finite elements mesh near the contact area. **b** Deformed shell in contact with gelatin phantom, stress distribution σ_*z*_ of the sample at *h* = 1.7 mm

The first stage of modeling is the same for all calculations and is independent of the choice of a sample. Deformation of the shell with applied hydrostatic pressure is considered as an axisymmetric problem. The shell has the shape of a hollow hemisphere with an attached cylindrical ring. The external radius of shell *R*_1_ is 5 mm with thickness *δ* of 0.4 mm. Length of the cylindrical part of the shell *l* is 4 mm (Fig. 6). External loads or displacements are preset on the inner surface of the cylindrical shell section. The origin *O* of the cylindrical coordinates system $Or{\varphi}z$ coincides with the shell center for the non-deformed condition, and the *Oz* axis goes up along the symmetry axis. Due to axial symmetry, the boundary conditions and the solution are independent of the angle coordinate *φ*.

Thus, the expressions below are written in the *rz* plane. Hydrostatic pressure *p*_*air*_, taking the value of *p*_1_ = 6 kPa or *p*_2_ = 15 kPa in the calculations, is applied to the inner surface of the shell:

1 $$p_{air} = p_{i}, \quad {\text{ i = 1,}}\; 2\quad {\text{for}}\;r^{2} + z^{2} = (R_{1} - \delta )^{2} .$$

The inner surface of the cylindrical part of the shell is attached to a metallic tube, which means that the elastic displacement vector component on that part of the shell surface is equal to zero:

2 $$u_{r} = u_{z} = 0,\quad r = R_{1} ,\quad z \in [0, \, l]$$

The shell is in an equilibrium state, with no body forces taken into account.

At stage 2, contact interaction of the deformed shell with the three samples is considered. Refer to the contact scheme in Fig. 6a. The contact problem is considered to be axisymmetric. The indenter and the silicone cylinder are located coaxially, and the surface area of the sample is much larger than the contact area. The initial contact of the shell and the sample takes place at a point located on the symmetry axis. The lower surface of the sample is fixed. Vertical displacements *h* are simulated on the cylindrical shell section, corresponding to the downward movements of the metallic tube, to which the silicone head is attached (Fig. 2). Horizontal and angular displacements of the cylinder are prohibited (*u*_*r*_ = 0) and the pressure *p*_*air*_ on the shell inner surface remains constant. Contact condition can be written in the following form:

3 $$u_{z}^{(1)} + u_{z}^{(2)} = h - f^{(1)} (r,z),\quad (r,z) \in \varOmega ,$$

where $u_{z}^{(i)}$, mm are elastic displacements at the contact area $\varOmega$, and $f^{(1)} (r,z)$ is the shape of the indenter. The normal and tangential stresses on the sample surface outside the contact area are equal to zero:

4 $$\sigma_{z} = \tau_{rz} = 0,\quad (r,z) \notin \varOmega .$$

The friction forces, calculated according to Coulomb’s law, are taken into account, and the friction coefficient *μ* can be specified for each contact pair:

5 $$\tau_{rz} = \mu \sigma_{z} ,\quad (r,z) \in \varOmega .$$

The values of friction coefficient *μ* are varied in ranges [0.3, 3] for PDMS and [0.1, 1] for the gelatin phantom.

The main contact parameters for calculation are the radius of the contact area and the parameter *u*_*s*_:

6 $$u_{s} = u_{z}^{(1)} (0,\, - R_{1} + \delta ) - u_{z}^{(1)} (R_{1} ,\;0 - h),$$

where *h* represents the value of the applied vertical displacements on the cylindrical section of the shell.

Both silicone and gelatin samples have cylindrical shape, with 6 mm radius and 12 mm height for silicone one, and 45 mm radius and 7 mm height for gelatin. The problem formulation may take into account friction between the sample and the base. However, calculations demonstrate that the friction, for the selected width of gelatin layer, negligibly affects the size of the contact area. We studied dependences of contact area on deformation of the sensor head for values of the friction coefficient from range ($0.1 \ldots 1$). Calculations show that difference in dependences is less than 2%.

To calculate the contact parameters and dependences of contact radius on load ANSYS 14.0 (Academic license) is used. The meshed fragment of the finite element model is shown in Fig. 6. Since the maximum contact area radius is about 3.5 mm, the uniform mesh is used for the contact zone and large neighbor area, and a free discretization with an increasing element size is used for the rest of the sample volume. Axisymmetric higher order 2-D, eight-node structural solid elements (plane 183) were used. A typical size of an element for the shell is 0.03 mm. The total number of elements for the sensor head and gelatin sample was 11,800 (for silicone case the number of elements is 11,000, for plastic case number is 5700). Calculations are conducted assuming large deformations of the objects, which allows taking into account the shape change in the process of deformation. The problem of identifying the elasticity modulus of gelatin is solved by minimizing the deviation of the computer simulation results from the experimental data. The dependences of contact area radius on the parameter *u*_s_ (“*a*-*u*_s_” curves) are calculated for Young’s modulus range from 140 to 260 kPa with an increment of 30 kPa to cover all the experimental data. The value of elastic moduli for gelatin phantom is chosen from the family of *a*-*u*_s_ curves.

## Results

The deformed shape of the indenter in contact with the gelatin sample is presented in Fig. 6b at *h* = 1.7 mm and inside pressure *p*_*air*_ = 6 kPa. Figure 6b shows vertical normal stress distribution σ_*z*_ of gelatin.

**Fig. 7:**
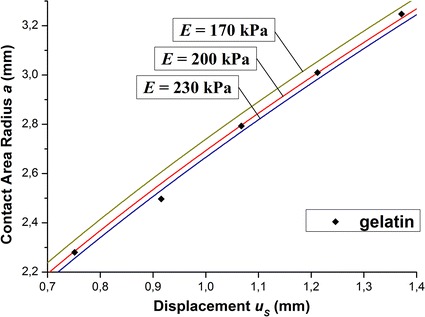
Approximation of experimental data for gelatin by calculated curves. Family of curves with 15% step (*E* = 200 ± 30 kPa)

After performing experiments the obtained data is depicted on the same plot and the standard deviation from the experimental data is calculated for each calculated curve (Fig. 7). The curve having the least standard deviation from the experimental data is chosen as the approximation curve for the current sample. The family of curves with such value of Young’s modulus step (±15%) is selected to assure that any set of experimental data could be approximated in this way by only one curve from the family.

**Fig. 8:**
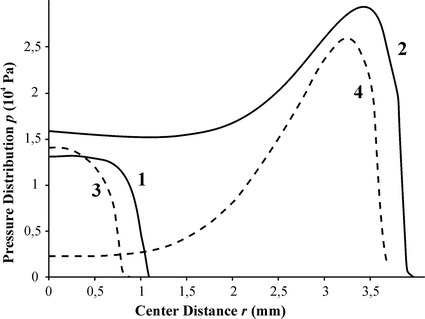
Distribution of contact pressure for gelatin phantom for various of *h*: 1.7 mm (curves *2*, *4*), 0.25 mm (curve *1*), 0.15 mm (curve *3*). *p*_*air*_ = 15 kPa (curves *1*, *2*) and 6 kPa (curves *3*, *4*)

**Fig. 9:**
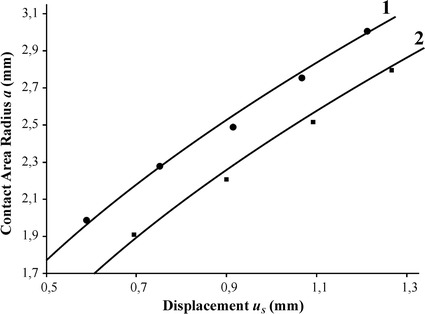
Dependence of the contact radius *a* on the displacement of the central point of the sensor shell *u*_*s*_ (experimental points and calculated curves) for tests with gelatin (*1*) and silicone (*2*)

From the finite element simulations, a distribution of contact pressure for different values of sensor indentation depth and additional pneumatic pressure was obtained (Fig. 8), as well as the dependence of the contact radius on parameter *u*_*s*_ (Fig. 9) for contact of the sensor shell with the silicone and gelatin phantom, respectively.

Distributions of the contact pressure for the sensor head during indentation of the gelatin phantom for three different depths and two interior pressure values are presented in Fig. 8. For *h* = 0.15 mm the contact pressure distribution is bell-shaped and it is similar to the pressure distribution in Hertz contact problem [15]. However, when the indentation depth increases to 1.7 mm, the maximum values of contact pressure are located near the edge of the contact area. For *h* = 1.7 mm, lower air pressure inside the shell results in lower contact pressure in whole contact area. The required minimal pressure from the air supply is 6 kPa which is enough to guarantee the circular shape of the contact area.

An analysis of the curves in Fig. 8 demonstrates that it is possible to control the effective stiffness of the sensor by adjusting the air pressure inside the shell. Under additional air pressure value *p*_*air*_ = 15 kPa (curve 2 in Fig. 8), the difference between the contact pressure in the center of the contact area and in the edge is not as high as for *p*_*air*_ = 6 kPa (curve 4).

From the experimental results, dependencies of *a* on *u*_*s*_ for samples with varied stiffness were obtained. A comparison of the calculation results and experimental data is shown in Fig. 9. The experimental results represent average values obtained in series of 5 tests with standard deviation less than 1%.

## Discussion

A comparison of the experiments and calculation results for the materials with known mechanical properties verified the calculation model. It is shown in calculations that the friction force has negligible effect on the dependence of the contact area size versus the parameter *u*_*s*_ for interaction with the glass, silicone and gelatin phantom.

The results show that the model simulation is consistent with the experimental data, and the mechanical properties of gelatin are well simulated by using an incompressible linear elastic model. Young’s modulus of gelatin phantom was determined using the least squares technique by fitting calculated curves with the experimental points and it was found to be 200 kPa. This value is close to elastic modulus estimations obtained for carcinoma [16], gelatin phantoms [17, 18], and porcine fat tissue [17]. It is necessary to notice that an error in determining the elasticity modulus of the material depends on its absolute value. For instance, if an elasticity modulus of tissue in consideration is comparable to modulus of the material of the sensor head, then the error in the elasticity modulus determination may be over 10% even when the calculated curve just deviated from experimental data with error less than 1%. For softer material, such as the gelatin phantom, errors of the elastic modulus determination are comparable with the deviation of the calculated curve from the experimental data (near 5%). Biological tissues have a wide range of elastic modulus, some for elastic moduli up to 1000 kPa (see, for example, [19]). The proposed sensor allows determination of effective Young’s modulus of tissues if this modulus is less than or equal to 200 kPa.

## Conclusion

The video-tactile pneumatic sensor allows observation of the contact area during the indentation process. The sensor and the methodology for determining elastic modulus of soft tissues ranged less than 200 kPa were developed. One of supplementary advantages of the sensor is the fact that the additional air pressure could be used to tune stiffness of the sensor and to increase its applicability for estimating elastic modulus of tissues within different ranges of values. The sensor can be used for stiffer materials if the additional pressure in the head will be increased. It was also demonstrated that mechanical behavior of phantom may be described by incompressible linear elastic body model.

We performed experiments with flat sample surfaces. Biological tissues have largely uneven surfaces. Before testing biological tissue the device should be oriented in such of way that the visible area of contact would be symmetrical about the central point.

Future prospects of the method could be as follows: application of the device in laparoscopic surgery, development of the device and theoretical model for tumor locating by generating a map of elasticity in a certain area, and improvement of existing laparoscopic instruments to provide force feedback during the operation.

The new sensor can be promising tool for determining elastic properties of tissues such as the liver during laparoscopic operations.

## Abbreviations

UMT: universal micro-mechanical tester
LED: light-emitting diode
PDMS: polydimethylsiloxane
